# Geospatial modeling of geographical spread of *Aedes* species, in relation to climatic and topographical factors in Lagos State, Nigeria

**DOI:** 10.1371/journal.pntd.0012860

**Published:** 2025-02-06

**Authors:** Ayodele Samuel Babalola, Adedapo O. Adeogun, Hala S. Thabet, Reham A. TagEldin, Tolulope Oyeniyi, Olanrewaju Adekunle, Romoke Izekor, Oluwakemi Adetunji, Olagundoye Olalekan, Ahmed Omotayo, Olakiigbe Abiodun, Adewale Daniel Adediran, Taye Adekeye, Adesoye O. Adegbola, Chidinma Isaac, Phillip O. Okoko, James F. Harwood

**Affiliations:** 1 Public Health and Epidemiology Department, Nigerian Institute of Medical Research, Yaba Lagos State, Nigeria; 2 United States Naval Medical Research Unit EURAFCENT, Cairo, Egypt; 3 Integrated Vector Management Department, National Malaria Elimination Program, Abuja, Nigeria; Universidade do Estado do Rio de Janeiro, BRAZIL

## Abstract

The ecology and biology of mosquito disease vectors of the genus *Aedes* are highly dynamic, adapting to various climatic and topographic factors which makes their control challenging. Evidence-based control of *Aedes* mosquitoes requires a detailed understanding of this adaptability, which is greatly influenced by environmental dynamics. Understanding the drivers of their distribution is hence pertinent to predict disease risk. To better understand drivers and dynamics, we studied the distribution of *Aedes* mosquitoes in Lagos State, Nigeria, and its connection to climatic and human factors. *Aedes* larvae and adults were collected from eight Local Government Areas (LGAs, four urban and four rural) in Lagos State, resulting in 98 occurrence points. Using 23 environmental variables, we modeled the geographic distribution of *Aedes* spp. under current climatic conditions. Human population density was overlaid to estimate the risk of arboviral diseases. Although *Aedes* mosquitoes were found in all the eight LGAs in different proportions, species distribution varied considerably. Both *Aedes aegypti* and *Aedes albopictus* were found across the LGAs with evidence of species partitioning. Virtually all the LGAs were predicted to be highly suitable environments for *Aedes* mosquitoes, with only two LGAs being moderately suitable. Anthropogenic factors including the extensive accumulation of tires contribute to larval habitat availability for both *Aedes aegypti* and *Aedes albopictus*. Urban areas with high population density were also associated with increased larval habitat availability when compared with rural areas. Furthermore, the model suggests that LGAs sharing border with Ogun State are highly suitable environments for *Aedes spp*. Our study highlights that the main contributing factors to *Aedes* distribution were precipitation and temperature in the coldest quarter. This paper aims to understand how human and climatic factors affect *Aedes* mosquitoes distribution in Lagos State, which is crucial to prevent disease transmission.

## Author summary

The adaptability of *Aedes* mosquitoes to diverse climatic and environmental conditions complicates their control, necessitating a deeper understanding of the factors driving their distribution to predict disease risk. This study examined the distribution of *Aedes* mosquitoes in Lagos State, Nigeria, using 98 occurrence points from eight Local Government Areas (LGAs), evenly divided between urban and rural regions. Ecological niche modeling with 23 variables revealed that precipitation and temperature during the coldest quarter were key drivers of distribution. Most LGAs were highly suitable for *Aedes* mosquitoes, with urban areas, high population density, and anthropogenic factors, such as tire accumulation and the presence of improperly disposed containers, contributing to increased larval habitat availability compared to rural areas. Both *Aedes aegypti* and *Aedes albopictus* were widespread but exhibited species partitioning. Additionally, some LGAs in Ogun State bordering Lagos were also highly suitable. These findings underscore the critical role of climatic and human factors in *Aedes* mosquito distribution, providing valuable insights for targeted disease prevention strategies in Lagos State.

## Introduction

Mosquito-borne diseases cause significant public health impact worldwide, and Nigeria is no exception to these issues of great epidemiological concerns. The prevalence of diseases, such as dengue, chikungunya, and Zika, primarily transmitted by *Aedes* mosquitoes, result in significant social and economic consequences in Nigeria [1]. For instance, in November 2020, an outbreak of Yellow Fever was reported in Delta State by the National Center for Diseases Control (NCDC). Among 48 suspected cases, there were 30 recorded deaths, resulting in a case fatality rate of 62.5% [2]. The occurrence and resurgence of such infectious diseases can be attributed to factors as urbanization and rapid human population growth, which lead to overcrowding, increased waste generation, and the creation of favorable environments for vectors. Recent reports indicate that about 367 out of 774 local government areas (LGAs) of Nigeria have reported yellow fever cases [3] with significant death rates [4]. Given the country’s diverse ecological landscapes, intricate climate patterns, and a burgeoning human population, controlling these vector-borne diseases remains a herculean task [5,6].

Nigeria’s vulnerability to mosquito-borne diseases stems from its geographical and climatic diversity, ranging from the arid northern savannah to the humid southern rainforests [7]. This ecological variance provides an abundance and diversity of larval habitats for different mosquito vectors. More recently, *Aedes aegypti* and *Aedes albopictus*, have risen to prominence as disease vectors in Nigeria, posing a substantial threat to public health [8]. Diseases such as dengue, chikungunya, and Zika have the potential to trigger widespread outbreaks with severe health and economic consequences.

*Aedes* mosquitoes are highly adaptable and can thrive in both urban and rural environments [9], thereby putting the entire nation at risk of arboviral infections. For example, in Lagos, DENV3 I genotype, which was considered relatively new to the country, has been reported to be actively circulating, and is largely responsible for most hospital admissions, especially among children under 5 and older adults [10]. In addition, both Zika virus and Chikungunya virus, which are transmitted by *Aedes* mosquitoes, have been reported in some parts of Nigeria, with seroprevalences of 19.2% and 64.9%, respectively. Although these diseases often present with mild symptoms, they can lead to severe complications, including neurological disorders and, in rare cases, fatalities [11–12]. Despite the escalating threat posed by *Aedes* mosquitoes in Nigeria, critical gaps in knowledge persist regarding their distribution, abundance, and the environmental factors influencing their prevalence [9]. Until recently, there was a dearth of comprehensive studies aimed at identifying the ecological intricacies of these vectors within the local context in Nigeria. Consequently, limited data were available to reinforce targeted intervention strategies and inform public health policies, directly impacting the transmission of vector-borne diseases.

To address these knowledge gaps, this study employs ecological niche modeling (ENM), using real time data to predict the distribution of *Aedes* mosquitoes in Lagos State, a densely populated urban center in West Africa. ENM integrates ecological and environmental data, enabling the development of predictive models for species distribution. It also offers a better understanding of the ecological requirements of vector species and the factors that determine their presence [13], forecasting the geographic distribution of disease vectors in regions with complex topographies and variable climatic conditions.

By predicting the occurrence of *Aedes* mosquitoes, ENM provides informative data that can be used to shape effective vector control strategies. Through the identification of high-risk zones and potential breeding sites offered by species modelling [8], public health authorities can prioritize interventions, encompassing measures such as insecticide and adulticide spraying systems, larviciding, and community engagement. ENM paves the way for a proactive and targeted approach to vector control, consequently mitigating the peril of arboviral outbreaks and their ensuing impacts. This study aims to address the knowledge gap, providing extensive information about the spatial distribution of *Aedes* mosquitoes and how climatic and human factors influence their distribution in Lagos State in Nigeria using maximum entropy modeling approach with recently collected empirical data. The maximum entropy (MaxEnt) modeling used in this study is a general-purpose method for making predictions or inferences from incomplete information [14,15]. This model is a presence-only modeling algorithm (i.e., absence data are not required). Additionally, its performance has been reported to be relatively better than other modeling methods in handling presence-only data [15,16]. The fact that the model is minimally influenced by small sample sizes while providing robust predictions, thereby ranking it among the top-performing modeling tools [16], made it the preferred choice for our study.

## Materials and methods

### Ethical approval

Ethical clearance for this study was obtained from the Ethics review committee, Nigerian Institute of Medical Research, Lagos. All methods including mosquito larval collection and breeding, laboratory analysis and data management were performed in accordance with the 1964 Declarations of Helsinki.

### Study area

**Fig 1:**
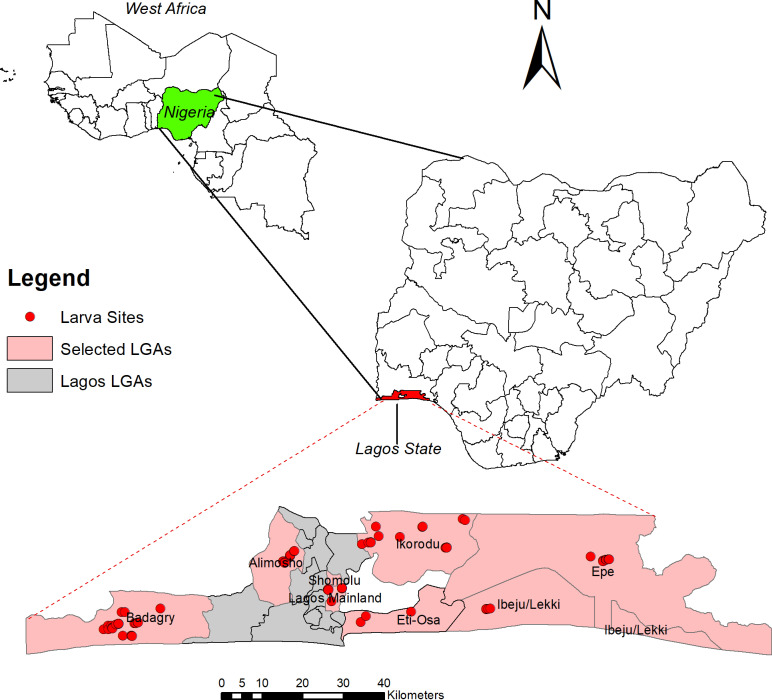
Map of Lagos State showing the sampling sites from eight selected LGAs. This figure was created by the authors in R programming software (R version 4.1.2, Vienna, Austria). Available at https://www.R-project.org/. The Nigerian shapefile was obtained from World Bank Data Catalog (https://data.humdata.org/dataset/geoboundaries-admin-boundaries-for-nigeria), an Open license standardized resource of boundaries (i.e., state, county) for every country in the world.

The study was carried out in Lagos State (6.5244° N, 3.3792° E), Nigeria. The state’s population is mostly concentrated on Lagos Island, in Lagos Lagoon, on the Bight of Benin in the Gulf of Guinea. Lagos is Nigeria’s largest city and one of the largest in sub-Saharan Africa. Lagos State has 20 LGAs with an estimated population of 15,946,000. The State is largely urban, though the recent classification still categorizes some of the LGAs as rural. The main method of vector control in the study areas is insecticide-treated bed nets (ITNs). In the past 5 years, ITNs have been deployed in the State through free mass distributions, resulting in high coverage (over 70%) of the areas. Generally, the climate in the State is typically tropical with two distinct seasons: rainy, from May to October, and dry, from November to April. The mean annual temperature ranges from 24.0^o^ C to 26.4° C, with a relative humidity that goes from 31.1% to 85% and average rainfall ranging from 1008mm to 1682mm. Larval collection was conducted in eight LGAs (Fig 1) spanning across different ecological zones (Table 1). The LGAs were selected to provide a balanced representation of both the ecological zones and the geographical areas across Lagos State.

**Table 1:** LGAs and ecological zones of sites where *Aedes* mosquitoes were collected.

Study LGAs	Ecological zone
Lagos Mainland	Mangrove Swamp & Coastal Vegetation and Freshwater Swamp Forest
Somolu	Freshwater Swamp Forest
Badagry	Mangrove Swamp & Coastal Vegetation
Ikorodu	Mangrove Swamp & Coastal Vegetation and Freshwater Swamp Forest
Ibeju/Lekki	Mangrove Swamp & Coastal Vegetation
Eti-Osa	Mangrove Swamp & Coastal Vegetation
Epe	Freshwater Swamp Forest
Alimosho	Freshwater Swamp Forest

### Mosquito collection and identification

The World Health Organization (WHO) and the U.S. Centers for Disease Control and Prevention (CDC)’s entomological protocols for field and laboratory studies on *Aedes* mosquitoes were used in this study [17]. Surveys of larval habitat sites were carried out in different communities across the eight LGAs (Table 1) and larval samples were collected between May and October 2022. Larvae were collected from potential habitats sites such as water filled tires, discarded containers, tree holes, discarded water tanks, car wrecks, unused water closets, and georeferenced using a Garmin eTrex 20.0 GPS device (Garmin International, Olathe, KS, USA). Collected larvae were transported to the insectaries for rearing under ambient laboratory conditions. Emerged adults were morphologically identified by two entomological technicians whose results were subsequently validated by a senior entomologist using the keys of Rueda [18] under a 1000x Dino-lite HD color CMOS sensor high speed digital microscope (model number, AD4113T-12V). To calculate the density of *Aedes* spp*.* across the LGAs, we used the Container index (Ci) as the percentage of water-holding containers with larvae and/or pupae [19,20].

### Environmental data

Climatic variables can have different impacts on species distribution. Variables such as temperature and precipitation influence species distributions at global and meso scales, while topographic variables such as altitude and aspect have more influence at meso and topo-scales. Finally, land-cover variables such as percent canopy cover can influence distributions at the micro-scale [8,15] and has been used in modeling the species of interest in another study [21]. Therefore, climatic, and topographic level variables were used to predict the distribution of *Aedes* mosquitoes in Lagos State and by extension to an adjoining State (Ogun State).

**Fig 2:**
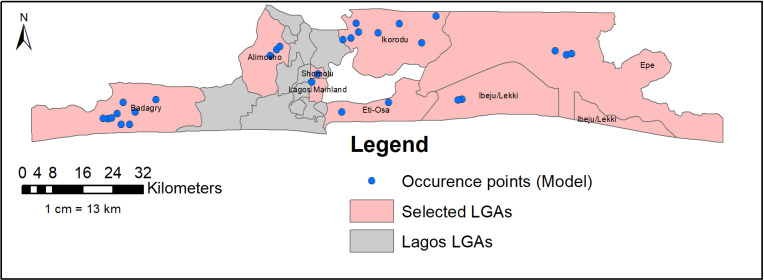
Forty unique occurrence points *of Aedes* mosquitoes in Lagos State were used for the model. This figure was created by the authors in R programming software (R version 4.1.2, Vienna, Austria). Available at https://www.R-project.org/. The Nigerian shapefile was obtained from World Bank Data Catalog (https://data.humdata.org/dataset/geoboundaries-admin-boundaries-for-nigeria).

A total of 82 out of 98 potential larval habitat locations were positive for *Aedes* mosquitoes across the eight LGAs. Autocorrelation problems were addressed by eliminating redundant values on the scale of the bioclimatic variables used in each 1 km × 1 km grid [22]. In addition, records for spatial autocorrelation were screened in ArcGIS 10.7.1 using average nearest neighbor analyses to remove spatially correlated data points [23,24]. After these adjustments, a total of 40 unique positive occurrence points for *Aedes* species were used in our prediction model (Fig 2). We considered 19 environmental and four topographical variables as potential predictors of the target species habitat distribution [25,26]. These variables were chosen based on their biological relevance to the target species distributions [8,21,26–28]. The nineteen bioclimatic variables with a 30 second spatial resolution (about 1 km^2^) were downloaded from the WorldClim database (http://www.worldclim.org/). The WorldClim data include 19 bioclimatic variables derived from monthly temperature and rainfall values collected at weather stations worldwide from 1950 to 2000 [29]. Elevation data at 1 km^2-^resolution were obtained from the Shuttle Radar Topography Mission (SRTM) and used to generate slope, aspect, and hillshade (all in degrees) with the Spatial Analyst tool/surface of ArcGIS 10.4.1 software.

The coordinates for all occurrence data were taken in decimal degrees (to four decimal places) and plotted using Google Earth to check for annotation errors. After downloading the climatic files, the Nigeria layer was extracted using a boundary mask, and the extracted files were converted to ASCII format using ArcGIS 10.7.1 software, and later used with MaxEnt software.

All combinations of the 23 environmental and topographic variables have been tested for multi-collinearity through the calculation of R-squared using the R software, ver. 4.1.2. Given that some of the bioclimatic variables were strongly correlated (R^2^ ≥ 0.7), only variables showing low correlation with other predictors were retained [28,30]. A total of 15 environmental and topographical variables were eventually selected (R^2^ < 0.7) (Table 2).

**Table 2:** Environmental variables used for modeling the potential distribution of *Aedes* spp.

No	Variables	Code/Unit	Source
1.	Annual Mean Temperature	Bio1	WorldClim
2.	Mean Diurnal Range	Bio2	WorldClim
3.	Max Temperature of Warmest Month	Bio5	WorldClim
4.	Mean Temperature of Driest Quarter	Bio9	WorldClim
5.	Mean Temperature of Warmest Quarter	Bio10	WorldClim
6.	Mean Temperature of Coldest Quarter	Bio11	WorldClim
7.	Precipitation of the Wettest Month	Bio13	WorldClim
8.	Precipitation Seasonality	Bio15	WorldClim
9.	Precipitation of Wettest Quarter	Bio 16	WorldClim
10.	Precipitation of Warmest Quarter	Bio 18	WorldClim
11.	Precipitation of the Coldest Quarter	Bio19	WorldClim
12.	Hill shade	–	SRTM
13.	Elevation	–	SRTM
14.	Aspect	–	SRTM
15.	Slope	–	SRTM

### Modelling

For this study, the preferred model was the maximum entropy (MaxEnt) modeling method [15,25,31,32] which is effective even with small sample sizes [26,30]. For the study area, MaxEnt only requires species presence data (not absence) and continuous or categorical environmental variable layers. We used the freely available MaxEnt software, version 3.3.3, which generates an estimate of the probability of the presence of the species that varies from 0 (“unsuitable”) to 0.99 (“best habitat suitability”) [15]. ASCII files of the 15 selected environmental variables and a CSV file of species presence coordinates, expressed in decimal degrees, were used to create the module. We utilized the ENMeval package (version 0.3.0) in R to identify the optimal settings for niche modeling using MaxEnt software [31]. A random cross-validation approach with 10 folds was applied for model evaluation. The Corrected Akaike Information Criterion (AICc) was employed to assess the complexity of the models [31]. Among the generated results, the model configuration with the lowest Delta AICc value was selected and implemented in MaxEnt (version 3.4.0) to generate the final model output.

MaxEnt’s performance was assessed using a threshold independent Receiver-Operating Characteristic (ROC) analysis and Area Under Receiver-Operating Characteristic Curve (AUC) values (values from 0.5 = random to 1 = perfect discrimination). The algorithm either runs 1000 iterations of these processes or continues until convergence is reached (threshold 0.00001).

The relative importance of each environmental predictor was evaluated using the percentage contribution of the Jackknife test, which is widely considered the most reliable index for small sample sizes [33]. The default logistic output format was then selected, indicating the probability of suitable conditions with values ranging from 0 to 1. A total of 75% of the location point data were used for training, and the remaining 25% to validate the predictive ability of the model. Average and standard deviation values for training and test AUC for the 40 replicates were extracted from the MaxEnt text result output. The ASCII output map for the average model for the target species was loaded in ArcGIS 10.7.1, where the prediction models of habitat suitability were divided into 5 classes: very low (0 – 0.1), low (>0.1 – 0.2), moderate (>0.2 – 0.4), high (>0.4 – 0.6) and very high (>0.6) [34,35].

## Results

### Larval Breeding Structures of *Aedes*, positivity rates and container index (CI) across 8 LGAs

Table 3 presents the total number of potential larval habitats encountered during the study in Lagos State. A total of 2,964 individual potential larval habitats were examined across the eight LGAs, with 65.1% (1,930 out of 2,964) testing positive for *Aedes* spp. The highest number of larval habitats was recorded in Ikorodu, followed by Lagos Mainland, while the lowest number was found in Somolu. Ikorodu also had the highest CI (91.7%), followed by Lagos Mainland (65.2%), while the lowest recorded CI was in Eti Osa (13.1%). It is noteworthy to mention that in Ikorodu and Lagos Mainland, LGAs with very high positivity rates, a higher number of discarded containers that served as larval habitat were retrieved.

**Table 3:** Total number of *Aedes* larval habitat encountered during the study.

LGAs	No. Examined	No. Positive	Container index (%)
Ikorodu	1348	1236	91.7
Epe	267	65	24.3
Ibeju Lekki	185	97	52.4
Badagry	167	64	38.3
Lagos Mainland	442	288	65.2
Somolu	124	53	42.7
Eti Osa	153	20	13.1
Alimosho	278	107	38.5
**TOTAL**	**2964**	**1930**	**65.1**

**Fig 3:**
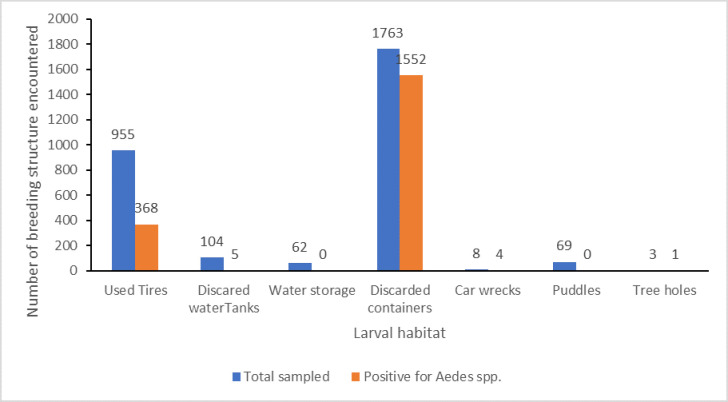
Types of larval habitat and their positivity for *Aedes* spp.

**Fig 4:**
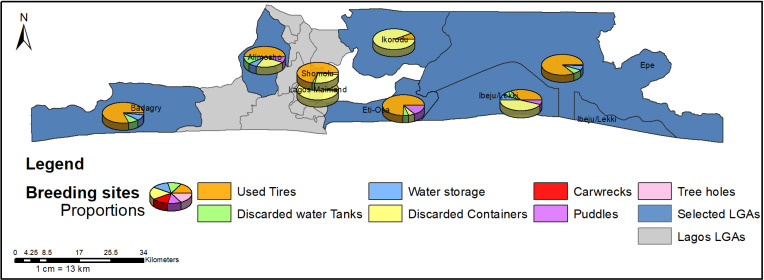
Proportion of different types of larval habitats encountered in the study. This figure was created by the authors in R programming software (R version 4.1.2, Vienna, Austria). Available at https://www.R-project.org/. The Nigerian shapefile was obtained from World BankDataCatalog (https://data.humdata.org/dataset/geoboundaries-admin-boundaries-for-nigeria).

A breakdown of the types of larval habitat is shown in Fig 3. The most frequently encountered larval habitat was discarded plastic containers, followed by used tires; consequently, discarded plastic containers and used tires had the highest positivity rates. Other positive sites encountered in this study, though with relatively low positivity, are discarded water tanks, car wrecks, and tree holes (Fig 3). Spatial distribution of each larval habitat is presented in Fig 4.

Table 4 reports the CI for *Aedes* mosquito larvae and pupae across the study locations. The results show that Ikorodu, Badagry, Lagos Mainland, and Alimosho have high indices, and that discarded containers have a higher CI across the LGAs, followed by used tires. Remarkably, all positive water-holding containers exceeded the WHO limits (CI ≤ 20) for potential dengue transmission [19,20].

**Table 4:** Container index for *Aedes* mosquitoes’ larvae across the study locations.

LGAs	Container index (%)						
	UsedTires	Discarded Tanks	Water Storage	Discarded plastic containers	Car wrecks	Puddles	Tree holes
Ikorodu	50.3	0.0	0.0	99.8	0.0	0.0	–
Epe	30.1	30.2	0.0	–	–	0.0	–
Ibeju Lekki	40.2	0.0	0.0	80.2	–	0.0	–
Badagry	50.1	0.0	0.0	–	–	0.0	100.0*
Lagos Mainland	60.0	0.0	–	70.4	100.0*	0.0	–
Somolu	40.2	0.0	–	50.3	–	–	0.0
Eti Osa	20.3	0.0	–	0.0	–	0.0	–
Alimosho	50.1	0.0	0.0	50.1	–	0.0	–

*Limited number of water-holding containers. Total car wrecks were 8 and total tree holes was 3. These numbers might not be sufficient to determine if the actual Ci for both containers is 100%.

### *Aedes* larval density for used tires and discarded containers

**Fig 5:**
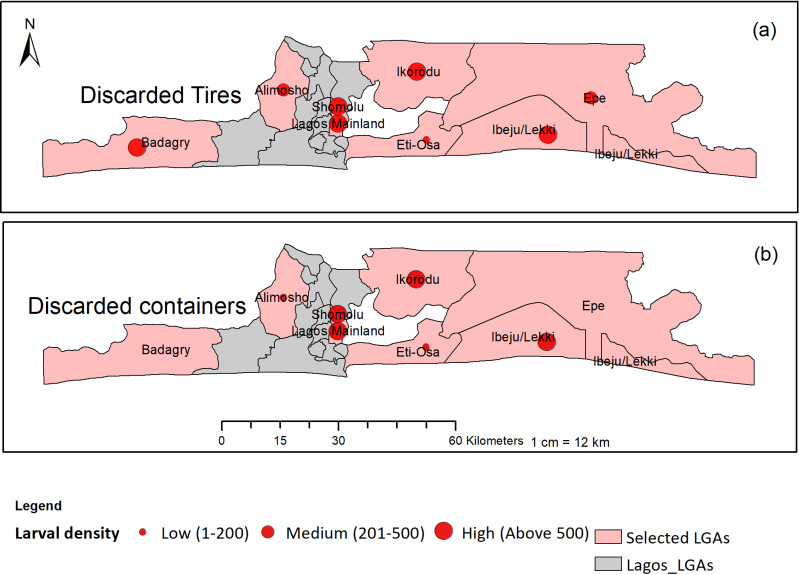
*Aedes* larval density for used tires and discarded containers. This figure was created by the authors in R programming software (R version 4.1.2, Vienna, Austria). Available at https://www.R-project.org/. The Nigerian shapefile was obtained from World BankDataCatalog (https://data.humdata.org/dataset/geoboundaries-admin-boundaries-for-nigeria) an Open license standardized resource of boundaries (i.e., state, county) for every country in the world.

The figure showing *Aedes* larval density for both used tires and discarded containers are presented in Fig 5a and 5b, respectively. The results showed that larval density ranged from low (1–200 larvae) to high (>500 larvae) per 10 scoops, per container. The results clearly showed that most of the LGAs recorded high larval density for both used tires and discarded containers.

### Spatial distribution of *Aedes* species

**Fig 6:**
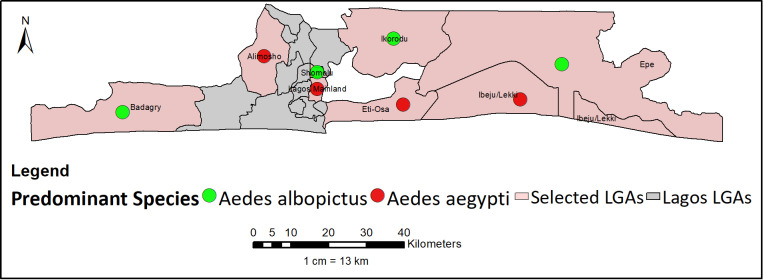
Predominant species of *Aedes* mosquitoes found in Lagos State. This figure was created by the authors in R programming software (R version 4.1.2, Vienna, Austria). Available at https://www.R-project.org/. The Nigerian shapefile was obtained from World BankDataCatalog (https://data.humdata.org/dataset/geoboundaries-admin-boundaries-for-nigeria) an Open license standardized resource of boundaries (i.e., state, county) for every country in the world.

*Aedes* mosquitoes were present in all the LGAs in varying proportions, exhibiting distribution variations. Both *Aedes aegypti* and *Aedes albopictus* were identified in water holding containers across LGAs with no sympatric occurrence of both species, indicating high level species partitioning. Specifically, *Aedes albopictus* were predominantly located in Badagry, Somolu, Ikorodu, and Epe, while *Aedes aegypti* were primarily concentrated in the remaining LGAs (Fig 6). Notably, all the *Aedes albopictus* specimens collected during this study were primarily situated in artificial containers, particularly plastic containers placed in shaded areas, often among clusters of banana plants. In contrast, the majority of the *Aedes aegypti* were discovered in used tires and discarded containers located in open areas, such as alongside roads, near office spaces, and around residential buildings. An estimated 1,920 abandoned tires and containers filled with water (with high positivity for *Aedes* spp.) were encountered across all eight LGAs. Regarding distribution across ecological zones, *Aedes aegypti* appears to be more prevalent in mangrove swamps and coastal vegetation, while *Aedes albopictus* shows a greater distribution in freshwater swamp vegetation.

### Potential habitat distribution for *Aedes* spp. in Lagos State

**Fig 7:**
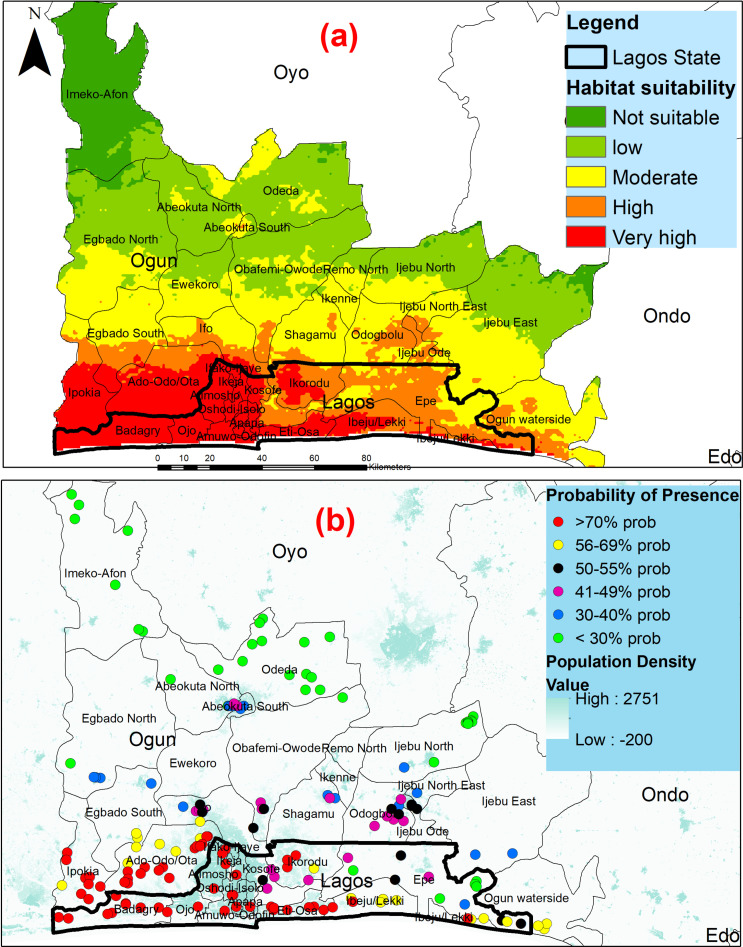
Predictive map of (a) *Aedes* mosquitoes distribution in Lagos and Ogun States (b) Overlay of population density with species distribution. This figure was created by the authors in R programming software (R version 4.1.2, Vienna, Austria). Available at https://www.R-project.org/. The Nigerian shapefile was obtained from World BankDataCatalog (https://data.humdata.org/dataset/geoboundaries-admin-boundaries-for-nigeria) an Open license standardized resource of boundaries (i.e., state, county) for every country in the world.

The potential habitat distribution of *Aedes* species is presented in Fig 7. The results reveal that suitable larval habitats for *Aedes* spp. are widely distributed across the State, albeit in varying proportions (Fig 7a). Virtually, all the LGAs were predicted to be highly suitable for *Aedes* mosquitoes, with the majority of them exhibiting very high suitability. Only Lagos Mainland and Epe presented some areas considered moderately suitable. Furthermore, the adjoining LGAs in Ogun State, specifically Ipokia,Ado-odo-ota, and areas in ten other neighboring LGAs, were also predicted to be highly suitable for *Aedes* mosquitoes. It is worth noting that the areas predicted to be highly suitable (with a probability of 70–99%) displayed high population density in both states, as depicted in Fig 7b.

### Model performance and influencing factors

The average percent contribution (PC) of the 15 variables used in the modeling of the *Aedes* spp*.* distribution was assessed. Precipitation of the wettest month had the highest contribution with a PC of 61.3%, followed by annual mean temperature with a PC of 12.3%, mean temperature of the coldest quarter (PC=8.4%) and finally precipitation of the coldest quarter with a PC of 6.2%. The results showed that these variables are strong predictors of *Aedes* distribution in the study locations, accounting for over 88% of the variations in the observed distribution (Table 5).

**Table 5:** Average percent contribution and permutation importance of the variables used in the modeling of *Aedes* species distribution in Lagos State.

Variables	Contribution (%)
Precipitation of the Wettest Month (bio13)	61.3
Annual Mean Temperature (bio1)	12.3
Mean Temperature of Coldest Quarter (bio11)	8.4
Precipitation of the Coldest Quarter (bio19)	6.2
Mean Temperature of Driest Quarter (bio9)	3
Mean Temperature of Warmest Quarter(bio10)	2.6
Precipitation of Wettest Quarter (bio16)	2
Precipitation of Warmest Quarter (bio18)	1.4
Hill shade	1.1
Precipitation Seasonality (bio15)	0.8
Max Temperature of Warmest Month (bio5)	0.5
elevation	0.3
aspect	0.2
Mean Diurnal Range (bio2)	0
slope	0

**Fig 8:**
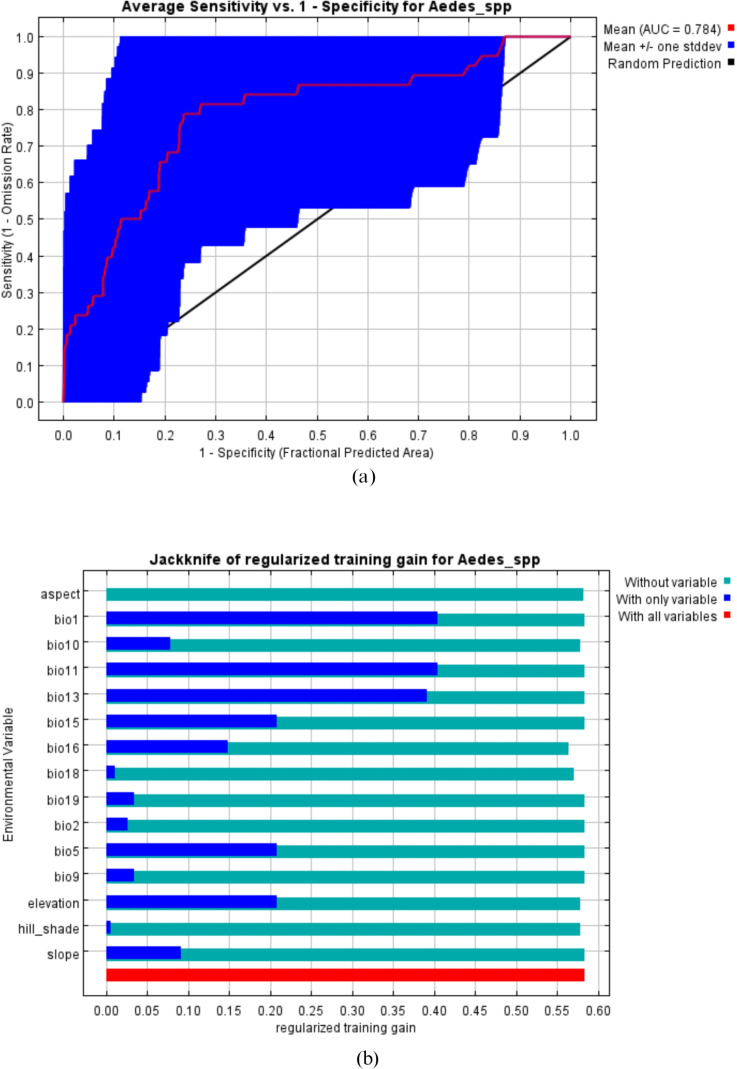
Estimation of model performance for *Aedes* spp. (a)Area under the curve (AUC) for *Aedes* species distribution. Red line indicates the mean value for 40 MaxEnt replicate runs. (b) Jackknife analysis for regularized training gain.

The ROC curve obtained as an average of the 40 replications runs is shown in Fig 8a. The average and standard deviation of the AUC for the 40 replicate runs was 0.784±0.254. This value indicates a very good performance of the model as an AUC value greater than 0.70 means high sensitivity and specificity for the presence of *Aedes* mosquitoes in the study area*.*

The relative importance of each variable was also validated with the Jackknife test (Fig 8b), providing a training gain of 0.6. The Jackknife test showed that annual mean temperature, mean temperature of the coldest quarter and precipitation of the wettest month are the three variables that will yield the highest increase in gain when used alone. The Jackknife test also showed that precipitation of the wettest quarter will slightly decrease the gain when removed from the model.

**Fig 9:**
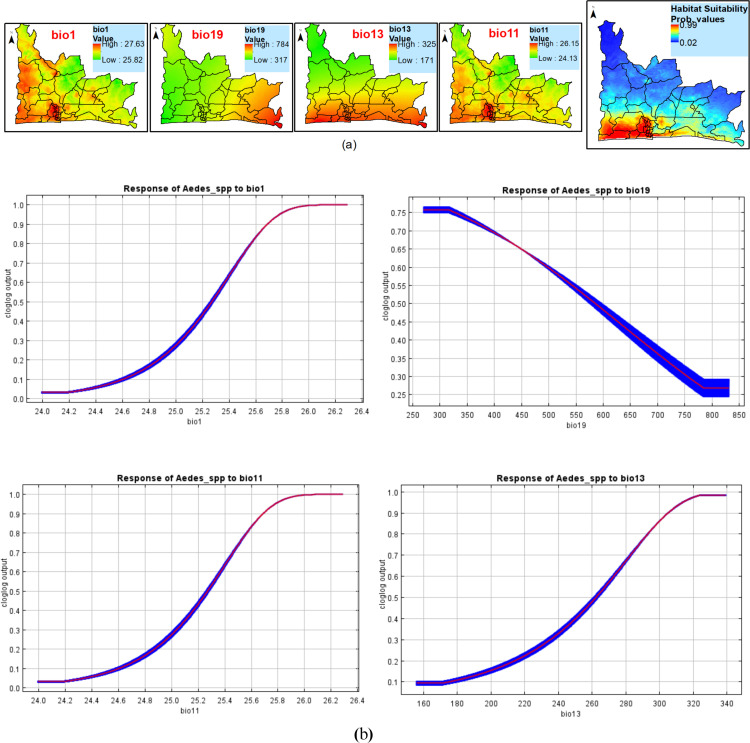
Estimates of the top contributing variables determining the geographical distribution of *Aedes* mosquitoes (a) The environmental variables anticipated to provide significant contributions to the geographical distribution of *Aedes* mosquitoes in Lagos State. Variable contributions (precipitation of wettest month, annual mean temperature, mean temperature of coldest quarter and precipitation of coldest quarter), (b) Response curves of four environmental predictors used in MaxEnt model for *Aedes* spp.

Fig 9 shows the variables providing the most significant contributions to *Aedes* spp. distribution in Lagos State. Spatial distribution analysis was performed to determine the geographical variability relative to the selected environmental variables in the State. The response curves of four variables to *Aedes* mosquito’s habitat suitability are shown in Fig 9a. The data obtained showed that annual mean temperature between 25.6 °C and 26.4 °C and precipitation during the wettest month ranging from 280 mm to 340mm favored the potential distribution of *Aedes* mosquitoes*.* Similarly, precipitation during the coldest quarter of 250 mm to 400 mm of rain significantly and potentially favored the distribution of *Aedes* mosquitoes. Finally, mean temperature during the coldest quarter ranging between 25.6 °C and 26.4 °C significantly favored the distribution of the *Aedes* mosquitoes in Lagos State (Fig 9b).

This figure was created by the authors in R programming software (R version 4.1.2, Vienna, Austria). Available at https://www.R-project.org/. The Nigerian shapefile was obtained from World BankDataCatalog (https://data.humdata.org/dataset/geoboundaries-admin-boundaries-for-nigeria) an Open license standardized resource of boundaries (i.e., state, county) for every country in the world

## Discussion

Vector surveillance is pivotal in arboviral disease control, facilitating early detection through the monitoring and identification of disease vectors. This approach empowers public health agencies to strategically deploy vector control interventions in high-risk zones, mitigating the risk of disease outbreaks. Furthermore, vector surveillance aids in the assessment of arboviral disease transmission potential, enabling proactive planning and resource allocation within impacted regions. Our work presents occurrence data for *Aedes* mosquitoes, primary vectors of dengue, yellow fever, chikungunya, and Zika, in eight LGAs of Lagos State. This study also provides predicted distribution for the entire state using the maximum entropy method.

To the best of our knowledge, there is no ongoing active surveillance for *Aedes* mosquitoes in Lagos State. Most of the surveillance efforts in Lagos and across Nigeria are primarily focused on malaria vectors. Consequently, because of lack of monitoring, these mosquitoes may have been undetected while spreading diseases. The available information on *Aedes* mosquitoes in Lagos has been largely carried out by individual researchers investigating insecticide resistance within the State, but their work only focused on *Aedes aegypti* [36,37]. Compared to previous studies, our work covers more sites and species, and, providing data about mosquito populations dynamics and distribution, has the potential to effectively highlight vector borne disease risk. The data gathered in this study can be important for policymakers and researchers to identify appropriate strategies to tackle the high disease risk that these vectors carry with them.

*Aedes* larvae were widely abundant in the study sites, particularly in high population densities. We demonstrated that the indiscriminate dumping of used tires and discarded containers significantly contributes to the widespread presence of these mosquitoes in Lagos State. This study reveals that all positive water-holding containers exceeded the WHO limits [18] for the container index (CI ≤ 20) across each LGA. This implies that residents of the LGAs stand at a high risk of a *Aedes*-vectored disease outbreak should there be a reservoir host in those areas. This observation may also help explain the resurgence of dengue virus infections in the state [10]. In the context of public health interventions, our findings emphasize the urgent need for targeted measures to work toward eliminating the identified larval habitats of *Aedes* mosquitoes. Implementing community-based initiatives to manage waste disposal, particularly focusing on used tires and discarded containers, have the potential to significantly reduce the mosquito population. Public health campaigns should also be implemented to raise awareness about the risk of dengue transmission associated with improper waste disposal practices.

Understanding the larval habitats of mosquitoes, their preferences for specific habitats, and the distribution of larvae are crucial factors for assessing risks and implementing effective mosquito control measures [38]. In our study, *Aedes aegypti* was predominant in mangrove swamps and coastal vegetation, while *Aedes albopictus* was more abundant in freshwater swamp vegetation. These data might have significant implications for mosquito control in Lagos State: differential habitat preferences suggest that targeted control measures should be employed based on the specific ecological zones. This finding underscores the significance of considering local variations in *Aedes* species composition, as targeting their ecological preferences can influence the success of control interventions [38].

In four of the eight LGAs surveyed, we found only *Aedes albopictus* and a previous study from these four LGAs reported only *Aedes aegypti,* [36], this on one hand might be due to the sites of collection (within the LGAs) which were different in the current study, indicating that both species may exist within microhabitats close to each other. On the other hand, this might suggest ongoing species displacement of *Aedes aegypti* by *Aedes albopictus* in these LGAs. *Aedes albopictus* is a highly invasive mosquito species that poses a substantial public health risk due to its vectoral competency for several arboviruses [39]. The ecological adaptability of *Aedes albopictus* facilitates its global expansion, primarily attributed to human mobility and the common practice of tire transportation. Nigeria, and particularly within the State of Lagos, are used as disposal sites for discarded tires from foreign countries. A key aspect of vector potential of *Aedes albopictus* is its inclination for urban and suburban environments, with larval habitat sites encompassing both natural and artificial containers [40]. However, it is documented that the ultimate prevalence of *Ae. albopictus* over *Ae. aegypti* in sympatric regions may be attributed to its elevated mating competitiveness [41]. These data may explain the reason for the ongoing suspected species displacement observed across the LGAs.

The increasing appearance of *Ae. albopictus* in these LGAs may signal a potential impending resurgence of arboviral diseases, notably yellow fever. There is mounting evidence of yellow fever resurgences in Nigeria [1,3], with *Aedes albopictus* establishment driving epidemiological changes and contributing to disease resurgence in Africa [42,43]. While several studies have reported the presence of *Aedes albopictus* in different parts of Nigeria [8,44–48], our findings are consistent with that of Anosike *et al.* [48] who recorded the presence of *Aedes albopictus* more frequently around Banana/Plantain plants in Imo State. The reason for this is not yet known as there is still no specific explanation for this scenario.

Our model indicates that nearly all LGAs are highly suitable for *Aedes* mosquitoes, with just two LGAs having moderately suitable areas. Mosquitoes’ adaptability is exemplified by their ability to endure harsh conditions. For instance, their eggs can remain dormant during the hot, dry season and hatch into larvae with the arrival of the rainy season. The larvae can even survive and mature to adulthood in a small water source, like a bottle cap, if a minimal amount of water is present. The fact that highly suitable environments for *Aedes* mosquitoes are found in densely populated areas is consistent with several studies reporting that the presence of these species is positively correlated with proximity to urban infrastructure and human population [49,50]. Another study mapping the global distribution of *Aedes* albopictus and *Aedes* aegypti reported a close association between population density and the occurrence of these species in a model-based estimate [28].

The distribution of disease vectors is influenced by complex relationships involving factors like climate, landforms, soil types, and human settlement patterns within various ecological zones [7,51,52]. However, this study specifically examines the impact of climatic and topographic elements on the distribution patterns of *Aedes* mosquitoes in different ecological zones in Lagos State. We found that precipitation during the wettest month has the highest influence on the distribution of *Aedes* spp., followed by the annual mean temperature and mean temperature of coldest quarter. This finding is consistent with another study from Enugu, Nigeria, which identified precipitation during the wettest month as the most important environmental variable in predicting *Aedes* mosquitoes, with a similar suitable precipitation range [8]. Additionally, studies from outside Nigeria have also recognized the leading role of rainfall in model-based estimates of habitat suitability for *Aedes* mosquitoes [28,53]. The ROC curve showed that colder climate supported the breeding of *Aedes* mosquitoes*,* which are more likely to be found in areas with higher precipitation during the wettest month of the year, making them highly prolific. Our model also indicated that low precipitation during the coldest quarter is more beneficial for the presence of *Aedes* species in Lagos State. This is in agreement with another study, which reported that excess rainfall during the coldest quarter of the year can flush out *Aedes* breeding sites, thereby reducing the mosquito population [21].

These findings underscore the significance of factoring in environmental variables, particularly climate and topography, when devising mosquito control strategies within these geographical contexts. Understanding mosquito species’ distribution and environmental preferences aids in pinpointing the timing and locations for deploying interventions like bed nets, indoor spraying, and larval management, particularly in high-risk regions. For instance, focusing on environmental modification efforts, such as proper disposal of unused containers, especially in areas with high precipitation during the wettest part of the year, can effectively control *Aedes* mosquito population. Furthermore, continuous monitoring and adaptive measures in response to environmental shifts and vector behavior can enhance the efficacy of arboviral diseases’ control efforts in the nation.

One significant limitation of this study is the exclusion of parameters such as pH, dissolved oxygen, total dissolved solids, electrical conductivity, turbidity of the breeding sites and demographic variables like the presence of cattle and socio-economic status from our model. These variables are also crucial and may contribute to the explanation of mosquito species distribution [6]. Further research should be conducted to incorporate these variables and evaluate their effects on the distribution of *Aedes* mosquitoes in the country at large.

Given that this study was conducted in a single state (Lagos State) in Nigeria, the external validity and generalizability of the findings may be limited. However, the predicted distribution patterns and environmental associations of *Aedes* mosquitoes identified in this study could provide valuable insights for similar urban areas with comparable climatic and ecological conditions. While extrapolation beyond Lagos State should be done with caution, the results can still inform vector control strategies in other densely populated, urban regions in Nigeria and potentially in West Africa with similar settings.

## Conclusion

Our study provides new evidence on *Aedes* mosquitoes- distribution in Lagos State, Nigeria. *Aedes albopictus* and *Aedes aegypti* are the two main vectors found in Lagos with high level species partitioning and possible displacement observed between *Ae. aegypti* and the more invasive *Ae. albopictus*. Specific environmental factors influence the distribution of these mosquitoes, requiring tailored control strategies. We have generated a model-based baseline species distribution of the *Aedes* mosquito population in Lagos using empirical data. Knowing that a state-wide mapping of vector distribution can be time consuming and expensive, the maps presented here could be used as a guide by the state ministry of health to direct resources for vector control within the state.
